# Prevalence of Brain Magnetic Resonance Imaging Diagnoses and Correlation With Signalment and Presenting Complaint in Dogs

**DOI:** 10.3389/fvets.2021.768709

**Published:** 2021-11-16

**Authors:** Nicholas Walsh, Patrick C. Carney, Shayna Streu, Margret Thompson, Philippa J. Johnson

**Affiliations:** ^1^Cornell University College of Veterinary Medicine, Cornell University, Ithaca, NY, United States; ^2^University of Missouri College of Veterinary Medicine, Columbia, MO, United States

**Keywords:** MRI, usage, breed, age, canine

## Abstract

Since magnetic resonance imaging (MRI) was introduced, it has become increasingly available and technologically improved. Studies have documented the prevalence of specific pathologies, however no previous veterinary studies have investigated the prevalence and distribution of pathology across all MRIs performed at a single institution. The present study aimed to evaluate the prevalence of MRI-diagnosed brain lesions and correlate these to patient signalment and presenting complaint. Archived MRI brain scans from 805 dogs were reviewed retrospectively. One board-certified veterinary radiologist at the institution retrospectively evaluated all reports to determine the most clinically pertinent imaging diagnosis for each case. Breed, age, and presenting complaint were obtained from the medical record for each patient. The most common imaging diagnoses across all dogs reviewed were no significant findings (35.16%), asymmetric encephalopathy or meningoencephalopathy (19.75%), and extra-axial intracranial mass (11.18%). Age of dogs differed by diagnosis (*p* <0.0001), with the median age of dogs diagnosed with a brain mass being greater than that of dogs with no significant findings and dogs with asymmetric encephalopathy or meningoencephalopathy (both *p* <0.0083). In dogs presenting with seizures, the odds of a brain mass increased with each additional year of age [*p* <0.0001, odds ratio 1.26 (95% CI 1.16–1.37)], whereas the odds of no significant finding [*p* <0.0001, OR 0.87 (0.82–0.93)] decreased. Our findings provide overview information on the types of disease observed in the clinical population and allow us to detect correlations between imaging diagnoses, presenting complaints, and signalment.

## Introduction

Magnetic resonance imaging (MRI) serves as a powerful diagnostic tool in veterinary medicine and is routinely utilized in neurology practices. The superior contrast resolution of MRI for soft tissue makes it the reference standard for evaluating the brain (1, 2). During neurological work-ups, reports generated by radiologists reading MRI studies provide imaging diagnoses which describe the intensity, structure, location, size and enhancement pattern of brain pathology. These imaging diagnoses, although often not definitive for a specific disease, help to narrow down the differential diagnoses, especially when combined with the patient's history, signalment, and clinical information (3). Although an imaging diagnosis of an intracranial mass in a dog with seizures could represent an abscess, hematoma, or neoplasia, being made aware of such a finding can still help in patient management and narrowing the differential diagnosis list. Likewise, a normal MRI can greatly influence which diagnoses are prioritized. Few disease processes can be diagnosed with high specificity based on MRI findings alone; therefore, taking into account the patient signalment and presenting complaint is important. Even for some of the most highly researched human neurologic conditions such as multiple sclerosis and Parkinson's disease, a definitive diagnosis cannot be made based on MRI findings alone (4, 5).

There is a need to better understand the prevalence of brain pathologies identified on MRI examinations. Understanding the prevalence of particular neuroimaging diagnoses and the associations between signalment and diagnosis can help guide patterns of MRI usage in patient populations, and provide the pretest probability of a given diagnosis as well as information about the interpretation of MRI findings. In human patients, this type of information has helped to clarify the frequency of incidental findings on brain MRI (6, 7) and to document the spectrum of lesions in patients with specific clinical signs like migraines (8).

The purpose of this retrospective study was to document the prevalence of MRI-diagnosed lesions within the canine brain at a single academic institution and determine how these relate to patient signalment and presenting complaint. This data provides an overview of what MRI diagnoses are commonly encountered in the academic veterinary hospital setting. Likewise, it characterizes the patient signalment associated with these specific imaging diagnoses, allowing us to evaluate associations between lesion type and patient age and breed. Having such information available may help guide clinicians in their recommendations to pursue advancing imaging.

## Materials and Methods

### Setting

This retrospective study was performed at an academic veterinary hospital analyzing patient data from October 10, 2011 to October, 10, 2018.

One Toshiba Vantage Atlas 1.5 Tesla MRI scanner (Toshiba Medical Systems, Tustin, CA) is located in the hospital and utilized for small animal, large animal, and research scans (purchased in 2009). The scanner has 10 integrated coil parts, 16-channel array electronics standard, and an actively shielded gradient coil (33 mT/m, SR 200). All patients were imaged under general anesthesia.

### Data Extraction

Data were retrieved retrospectively from the hospital electronic medical record and picture archiving and communication system (PACS), tabulated using a custom in-house data retrieval system, and uploaded to Microsoft Excel 2016 (Microsoft, Redmond, WA, USA). Data extracted for each study included: species, breed, age, presenting complaint, and imaging diagnoses.

One board-certified veterinary radiologist at the veterinary hospital retrospectively evaluated previously completed imaging reports for all canine brain examinations to determine the most clinically pertinent imaging diagnosis. The majority of reports had multiple findings, but the single most pertinent diagnosis was determined based on reason for presentation and subjective clinical interpretation. Histologic diagnoses were not included as such information was not available for a large proportion of cases. It is important to note that an imaging diagnosis is a descriptive term only and may represent multiple different underlying causes. For example, an intracranial extra-axial mass lesion may represent any disease process that would create this characteristic imaging finding, such as a meningioma, histiocytic sarcoma, hematoma or abscess.

Specific imaging diagnoses were categorized based on the following criteria. For brain diagnoses, “no significant findings” (NSF) indicated that no abnormalities were identified to explain the presenting complaint. Mass lesions were divided into intracranial extra-axial, intra-axial, suprasellar and intraventricular anatomic categories. The imaging diagnosis of encephalopathy was used for any gray or white matter parenchymal lesion that was a mass, cavity, hemorrhage or malformation. An example would be ill-defined T2-weighted hyperintensity secondary to inflammatory brain disease. Encephalopathies were a presumed diagnosis and were divided according to the distribution of lesions and included asymmetric encephalopathy or meningoencephalopathy (AEM), symmetric encephalopathy, diffuse encephalopathy or meningeal thickening. Extracranial lesions were divided into those with and without intracranial extension. Other categories included cyst/fluid cavitations, brain malformations, ventriculomegaly, intracranial hemorrhage, presumed cerebellar atrophy or abiotrophy, age-related changes (cerebral atrophy and/or microbleeds), infarcts, caudal occipital malformation syndrome (COMS), and cranial nerve lesions. It should be re-iterated that these are imaging diagnoses and presumed diagnoses, histopathological diagnoses were not utilized.

### Data Analysis

Categorical variables were summarized by frequencies and percentages. Continuous variables were assessed for normality via the Shapiro-Wilk test; those with approximately normal distributions were summarized by the mean and standard deviation, while non-normally distributed data were summarized by median, interquartile range (IQR), and range. Cases which lacked a recorded value for a given variable were not included in the relevant analysis but were included in all other data calculations. For summary statistics, patient age was divided into three age categories (juvenile, adult, senior); age categories were defined as: juveniles, 0–1 year; adults, >1–9 years; and seniors, >9 years (9). For patients with more than one presenting complaint (seizures, altered mentation, etc.), all were included in the calculations, resulting in more presenting complaints than the number of patients.

Statistical comparisons involving categorical variables were performed via the Chi square test; if the expected value of any cell was <5, Fisher's exact test was used. *Post-hoc* analysis employed Bonferroni correction. Relative risks and associated 95% confidence intervals were calculated for categorical comparisons, with confidence intervals adjusted for multiple comparisons. Relative risk point estimates indicating a >10% change in risk vs. the comparison group were reported regardless of statistical significance. For approximately normally distributed continuous independent variables with two groups, a two-sample *t*-test was used, while non-normally distributed data were assessed via the Wilcoxon rank sum test. For approximately normally distributed continuous independent variables with more than two groups, an ANOVA was performed, with Tukey's test for *post-hoc* comparisons; for non-normally distributed variables with more than two groups, the Kruskal-Wallis test was used, with *post-hoc* comparisons via Dunn's test. Odds ratios and associated 95% confidence intervals were calculated via logistic regression for both univariate and multivariate associations; Firth's penalized likelihood was utilized when pseudo-separation was noted.

Confidence intervals that did not include the null percentage were considered to differ significantly from the expected distribution. All statistical analyses were conducted using commercially available statistical software (SAS 9.4, SAS Institute, Cary, NC, USA).

## Results

During the study period, 805 scans of the brain were performed in canines for clinical purposes.

### Signalment

#### Age

The median age of dogs undergoing brain scans was 7.01 years (IQR 5.66, range 0.17–17.00). 63.7% of canine brain imaging patients were in the adult category, with 7.2% juveniles and 29.1% seniors. The distribution of imaging diagnoses within each of the three age categories is presented in Table 1. For the three most common MRI diagnoses (NSF, AEM, and intracranial mass) the age of dogs differed by diagnosis (Kruskal-Wallis *p* < 0.0001), with the median age of dogs diagnosed with a brain mass (8.52 years, IQR 3.2, range 0.7–15.5) being greater than that of dogs with no significant findings (6.0 years, IQR 5.9, range 0.2–14.1) and dogs with AEM (5.8 years, IQR 5.0, range 0.3–14.0) (Dunn's test, both multiple comparison-adjusted *p* < 0.05).

**Table 1:** Distribution [number (*n*) and percentage (%)] of most pertinent canine brain imaging diagnoses for all dogs and within each age category.

**Most pertinent imaging diagnosis**		**All dogs**	**Juvenile**	**Adult**	**Senior**
NSF	*n*	283	24	197	62
	%	35.16	41.38	38.40	26.50
Presumed encephalopathy (all)	*n*	203	15	152	36
	%	25.22	25.86	29.63	15.38
AEM	*n*	159	13	119	27
	%	19.75	22.41	23.20	11.54
Symmetric encephalopathy	*n*	19	1	16	2
	%	2.36	1.72	3.12	0.85
Diffuse encephalopathy	*n*	10	0	8	2
	%	1.24	0	1.56	0.85
Meningeal thickening	*n*	15	1	9	5
	%	1.86	1.72	1.75	2.14
Intracranial mass lesion (all)	*n*	180	2	102	76
	%	22.36	3.45	19.49	33.33
Extra-axial	*n*	90	0	48	42
	%	11.18	0	9.36	17.95
Intra-axial	*n*	59	1	38	20
	%	7.33	1.72	7.41	8.55
Suprasellar	*n*	24	0	8	16
	%	2.98	0	1.56	6.84
Intraventricular	*n*	7	1	6	0
	%	0.87	1.72	1.17	0
Extracranial lesion (all)	*n*	28	0	17	11
	%	3.48	0	3.31	4.70
with intracranial extension	*n*	9	0	4	5
	%	1.12	0	0.78	2.14
without intracranial extension	*n*	19	0	13	6
	%	2.36	0	2.53	2.56
Age related brain changes	*n*	21	0	4	17
	%	2.61	0	0.78	7.26
Ventriculomegaly	*n*	18	10	7	1
	%	2.24	17.24	1.36	0.43
Infarct	*n*	17	0	7	10
	%	2.11	0	1.36	4.27
Cranial nerve lesion	*n*	14	0	11	3
	%	1.74	0	2.14	1.28
Cyst or fluid filled cavitations	*n*	14	2	8	4
	%	1.74	3.45	1.56	1.71
Intracranial hemorrhage	*n*	11	0	2	9
	%	1.37	0	0.39	3.85
Brain malformation	*n*	34	15	15	4
	%	4.30	26.32	2.96	1.76
Presumed cerebellar atrophy or abiotrophy	*n*	5	2	2	1
	%	0.62	3.45	0.39	0.43
COMS	*n*	5	0	3	2
	%	0.62	0.00	0.58	0.85
Total	*n*	805	58	513	234
	%	100	100	100	100

*This table only includes imaging diagnoses with ≥5 cases. “NSF” indicated that no abnormalities were identified to explain the presenting complaint. “Age related brain changes” include atrophy and microbleeds. Canine age categories were defined as: juveniles, 0–1 year; adults, >1–9 years; and seniors, >9 years. AEM, asymmetric encephalopathy or meningoencephalopathy; COMS, caudal occipital malformation syndrome; NSF, no significant findings*.

#### Canine Breed

One hundred sixteen canine breeds (including mixed breeds) were represented. The prevalence of imaging diagnoses within common canine breeds is presented in Table 2.

**Table 2:** Distribution [number (*n*) and percentage (%)] of select canine brain imaging diagnoses by selected breed.

		**NSF**	**Extra-axial**	**Intra-axial**	**Suprasellar**	**IV**	**AEM**	**Symmetric**	**Diffuse**	**CN**	**Infarct**	**Other**
			**mass**	**mass**	**mass**	**mass**		**enceph**	**enceph**	**lesion**		
**All scans (*n* = 805)**	***n***	**283**	**90**	**59**	**24**	**7**	**159**	**19**	**10**	**14**	**17**	**123**
	**%**	**35.16**	**11.32**	**7.30**	**2.98**	**0.87**	**19.75**	**2.36**	**1.24**	**1.74**	**2.11**	**15.28**
Mixed breed (*n* = 185)	*n*	75	19	11	10	0	24	7	2	1	3	33
	%	40.54	10.27	5.95	5.41	0	12.97	3.78	1.08	0.54	1.62	17.84
Labrador ret. (*n* = 54)	*n*	17	13	1	1	1	12	0	0	3	2	4
	%	31.48	24.07	1.85	1.85	1.85	22.22	0	0	5.56	3.70	7.41
Golden ret. (*n* = 39)	*n*	17	8	3	1	0	6	0	0	0	0	4
	%	43.59	20.51	7.69	2.56	0	15.38	0	0	0	0	10.26
Boxer (*n* = 32)	*n*	7	2	13	1	2	3	0	0	0	1	3
	%	21.89	6.25	40.63	3.13	6.25	9.34	0.00	0.00	0.00	3.13	9.34
Boston terrier (*n* = 26)	*n*	5	0	5	3	0	8	1	0	0	1	3
	%	19.23	0	19.23	11.54	0	30.77	3.85	0	0	3.85	11.54
Pug (*n* = 26)	*n*	7	1	0	0	0	10	1	0	0	0	7
	%	26.92	3.84	0	0	0	38.46	3.84	0	0	0	26.92
Yorkshire terrier (*n* = 24)	*n*	3	3	1	0	1	10	0	0	0	1	5
	%	12.50	12.50	4.17	0	4.17	41.67	0	0	0	4.17	20.83
Dachshund (*n* = 20)	*n*	8	2	0	1	0	8	0	0	0	0	1
	%	40.00	10.00	5.00	4.76	0.00	40.00	0.00	0.00	0.00	0.00	5.00
Chihuahua (*n* = 18)	*n*	5	1	1	0	0	3	1	1	0	0	6
	%	27.78	5.56	5.56	0	0	16.67	5.56	5.56	0	0	33.33
Shih tzu (*n* = 18)	*n*	3	0	0	0	0	7	0	2	1	1	4
	%	16.67	0	0	0	0	38.89	0	11.11	5.56	5.56	22.22
Maltese (*n* = 18)	*n*	4	0	0	0	0	10	1	1	0	0	2
	%	22.22	0	0	0	0	55.56	5.56	5.56	0	0	11.11
GSD ( *n* =15)	*n*	7	1	0	0	0	3	1	0	0	0	3
	%	46.67	6.67	0	0	0	20.00	6.67	0	0	0	20.00
French bulldog (*n* = 14)	*n*	4	1	3	0	0	2	0	0	1	0	3
	%	28.57	7.14	21.43	0	0	14.29	0	0	7.14	0	21.43
Border collie (*n* = 11)	*n*	5	2	0	1	0	1	1	0	0	0	1
	%	45.45	18.18	0	9.09	0	9.09	9.90	0	0	0	9.09

*Only the commonly scanned canine breeds and select imaging diagnoses were included. All imaging diagnoses not listed were grouped under the “other” category. “NSF” indicated that no abnormalities were identified to explain the presenting complaint. AEM, asymmetric encephalopathy or meningoencephalopathy; IV, intraventricular; Enceph, encephalopathy; CN, cranial nerve; GSD, German Shepherd Dog*.

### Presenting Complaint

Five hundred fifty two dogs (68.6%) had one presenting complaint listed, 187 (23.2%) had two, and 53 (6.6%) had three or more; 13 (1.6%) had no presenting complaint listed. Seizures and abnormal mentation were seen in 23 patients, while seizures and behavior change were noted in 10. No other combination of clinical signs occurred in more that 10 patients. Seizures, abnormal mentation, and vestibular signs were the most common presenting complaints in dogs. Global testing indicated a significant difference (*p* = 0.0359) between the median ages of dogs with the three most common presenting complaints of abnormal mentation (15.0%), vestibular signs (20.8%), and seizures (38.8%), with *post-hoc* testing finding that dogs presenting with vestibular signs (median 7.4 years, IQR 4.8, range 0.7–15.3) were significantly older than those presenting for seizures (7.0 years, IQR 5.9, range 0.2–17.0; Dunn's test multiple comparison-adjusted *p* < 0.05) but not significantly different from those with altered mentation (6.8 years, IQR 6.2, range 0.3–14.0).

#### Seizures

**Figure 1:**
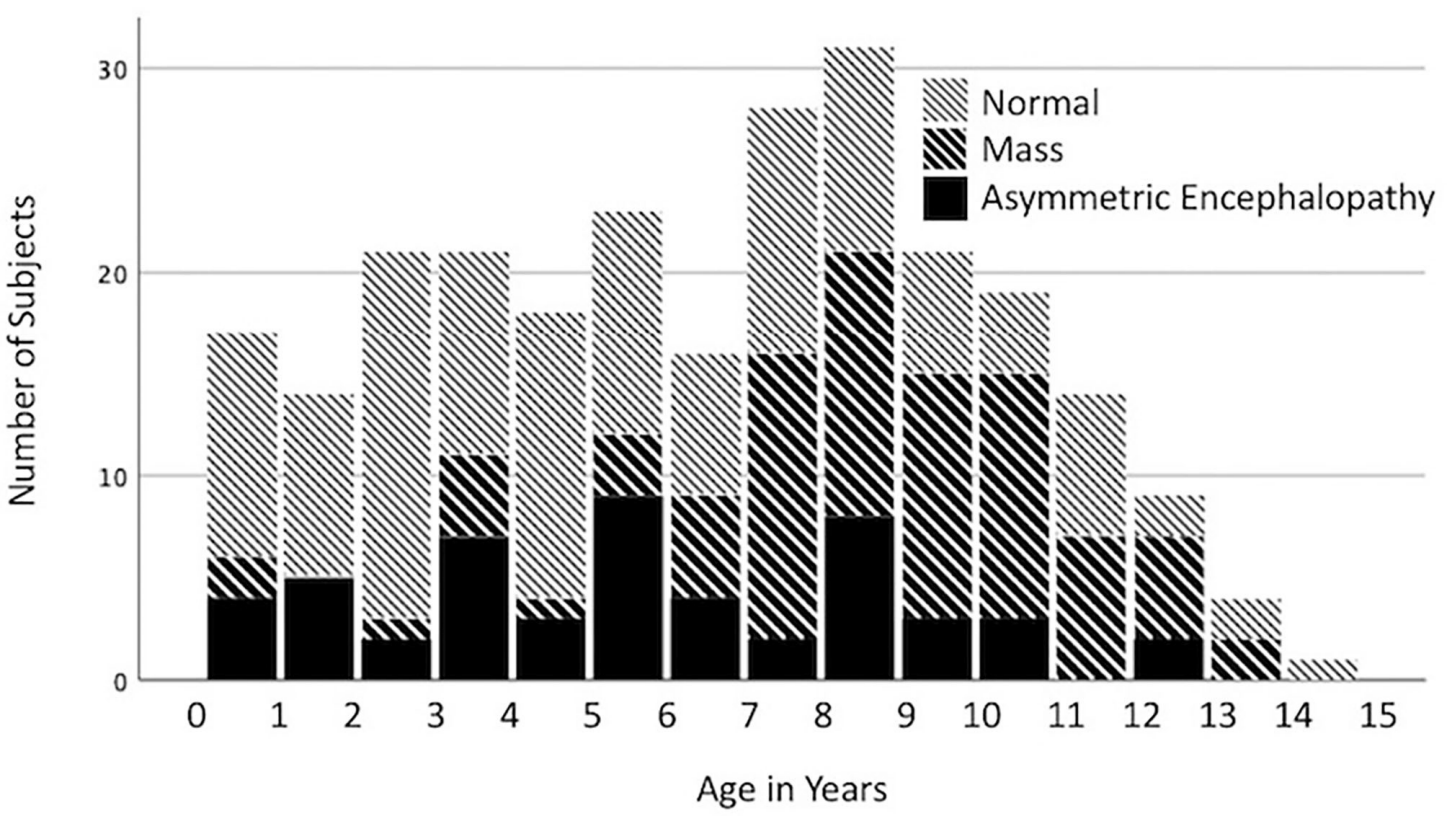
Distribution of the imaging diagnoses, no significant findings (NSF), mass, and asymmetric encephalopathy or meningoencephalopathy (AEM) by age for dogs presenting with seizures.

Among all dogs (*n* = 312; 38.8%) that presented for seizures, the most common diagnoses were NSF (39.4%), intracranial mass (26.0%) and AEM (16.7%). In dogs presenting with seizures, the odds of a brain mass diagnosis increased with each additional year of age [*p* < 0.0001, odds ratio 1.26 (95% CI 1.16–1.37)], whereas the odds of NSF [*p* < 0.0001, OR 0.87 (0.81–0.93)] decreased (Figure 1). No significant effect was found for age on the diagnosis of AEM [*p* = 0.0853, OR 0.93 (0.85–1.01)]. In multivariate logistic regression, neither breed (*p* = 0.2879) nor age (*p* = 0.1355) were found to be predictive of seizures as a presenting complaint.

#### Altered Mentation

One hundred twenty one dogs (15.0%) presented with altered mentation, of which 21 (17.4%) had a diagnosis of NSF. The most common imaging diagnoses were brain mass [28.9% (*n* = 35)] and AEM [27.3% (*n* = 33)]. In dogs with abnormal mentation, the odds of a brain mass diagnosis increased with each additional year of age [OR = 1.13 (95% CI 1.02–1.27)]. In multivariate logistic regression, breed (*p* = 0.0338) but not age (*p* = 0.7327) was found to be predictive of altered mentation as a presenting complaint, with shih tzus possibly having increased risk [*p* = 0.0504, OR = 2.75 (95% CI 1.00–7.60)].

#### Vestibular Signs

One hundred sixty seven dogs (20.7%) presented with vestibular signs. The most common imaging diagnoses for dogs with this presentation were NSF [30.5% (*n* = 51)], AEM [25.8% (*n* = 43)] or brain mass [22.2% (*n* = 37)]. In contrast to dogs with seizures or abnormal mentation, age did not significantly impact the odds of brain mass diagnosis in dogs with vestibular signs [OR 1.05 (95% CI 0.95–1.16)]. Age was significantly inversely associated with the odds of a diagnosis of AEM in dogs with vestibular signs [*p* < 0.0001, OR 0.78 (0.70–0.87)]. Age but not breed was significantly associated with vestibular signs, with an 8% increase in the odds of vestibular signs per year (*p* = 0.0151, OR = 1.08 (95% CI 1.02–1.15)].

### Brain Imaging Diagnoses

The most common imaging diagnoses were NSF (35.16%), AEM (19.75%), and extra-axial intracranial mass (11.18%) (Table 1).

#### No Significant Findings

This was the most common imaging diagnosis for brain MRI in dogs. The imaging diagnosis of NSF had the highest prevalence among juveniles (41.38% of juvenile dogs) (Table 1). Within breed groups, NSF was the most prevalent diagnosis in German shepherds (46.67%), border collies (45.45%), golden retrievers (43.59%), and mixed breeds (40.54%) (Table 2). The most common presenting complaints for dogs with NSF on brain scans were seizures [43.46% (*n* = 121)] and vestibular signs [18.02% (*n* = 51)].

#### Brain Mass

For canine brain scans, 22.36% had some form of intracranial mass as the most pertinent imaging diagnosis. These included 50.0% extra-axial, 32.78% intra-axial, 13.33% suprasellar and 3.89% intraventricular. The frequency of intracranial brain tumors varied widely between breeds (Table 2); in breeds with at least 10 subjects represented, the frequency ranged from 0% in Maltese and shih tzus, to 56.3% of boxers. Multivariate logistic regression incorporating age and breed found both to be significant predictors of the presence of an intracranial mass, with boxers having a 5-fold increase the odds of a brain mass compared to mixed-breed dogs and with a 21% increase in the odds for each additional year of age (Table 3). In boxers and Boston terriers, the mass lesions were commonly intra-axial, whereas in Labrador and golden retrievers they were commonly extra-axial [relative risk of intra-axial tumors for Boston terriers and boxers compared to Labrador and golden retrievers 7.22 (95% CI 2.57–20.26)]. The most common presenting complaints for dogs with brain mass lesions were seizures [45.0% (*n* = 81)], altered mentation [19.4% (*n* = 35)] and vestibular signs [20.60% (*n* = 37)].

**Table 3:** Breed-specific age-adjusted odds ratios and associated 95% confidence intervals for diagnosis of an intracranial mass in dogs undergoing brain imaging.

**Variable**		***P*-value**	**Odds**	**95% confidence**
			**ratio**	**interval**
Breed	Border collie	0.4899	1.66	0.39–7.00
	Boston terrier	0.3097	1.64	0.63–4.22
	Boxer	<0.0001	5.08	2.25–11.47
	Cavalier King Charles spaniel	0.2534	0.17	0.01–3.50
	Chihuahua	0.3055	0.47	0.11–1.99
	Dachshund	0.7919	0.84	0.22–3.17
	French bulldog	0.1104	2.80	0.79–9.89
	German Shepherd Dog	0.8626	0.88	0.20–3.85
	Golden retriever	0.2877	1.54	0.70–3.40
	Labrador retriever	0.2934	1.47	0.72–3.02
	Maltese	0.1982	0.15	0.01–2.74
	Pug	0.0716	0.20	0.03–1.15
	Shih tzu	0.1322	0.10	0.01–1.99
	Staffordshire bull terrier	0.7399	1.26	0.33–4.80
	Yorkshire terrier	0.9612	1.03	0.36–2.97
	Mixed breed	–	1.00 (REF)	–
Age		<0.0001	1.21	1.13–1.29

*REF, reference group*.

#### Encephalopathy

Some form of presumed encephalopathy was the most pertinent imaging diagnosis for 25.2% of all canine brain scans, with AEM comprising 78.3% of all presumed encephalopathies and being the most frequent form across all age groups. The frequency of AEM in dogs undergoing brain imaging differed significantly between breeds; in breeds with at least 10 subjects, the highest incidence of AEM was observed in Maltese (55.6%), Yorkshire terriers (41.7%), dachshunds (40.0%), shih tzus (38.9%), and pugs (38.5%), compared to 17.0% in all other breeds (Chi square *p* < 0.0001; Table 2). In multivariate modeling, increased age reduced the odds of a diagnosis of AEM [odds ratio for a 1 year increase in age 0.87 (95% CI 0.81–0.93)]; breed remained a significant predictor (*p* < 0.0004; Table 4). Dogs with an imaging diagnosis of AEM most commonly presented for seizures (32.7%), vestibular signs (27.0%), and altered mentation (20.8%).

**Table 4:** Breed-specific age-adjusted odds ratios and associated 95% confidence intervals for diagnosis of asymmetric encephalopathy/encephalomyelopathy in dogs undergoing brain imaging.

**Variabe**		***P*-value**	**Odds**	**95% confidence**
			**ratio**	**interval**
Breed	Border collie	0.5107	1.05	0.16–6.79
	Boston terrier	0.1848	3.36	1.28–8.80
	Boxer	0.1751	0.87	0.26–2.93
	Cavalier King Charles spaniel	0.1478	0.24	0.01–4.76
	Chihuahua	0.9192	1.76	0.48–6.43
	Dachshund	0.0360	5.76	1.81–18.28
	French bulldog	0.3472	0.97	0.22–4.29
	German Shepherd Dog	0.4331	1.09	0.25–4.76
	Golden retriever	0.6091	1.49	0.57–3.94
	Labrador retriever	0.6853	2.16	0.99–4.73
	Maltese	0.0055	7.11	2.52–20.06
	Pug	0.0432	4.41	1.75–11.12
	Shih tzu	0.0868	4.33	1.50–12.48
	Staffordshire bull terrier	0.5662	1.25	0.28–5.59
	Yorkshire terrier	0.0145	5.39	2.10–13.89
	Mixed breed	–	1.00 (REF)	–
Age		<0.0001	1.21	0.81–0.93

*REF, reference group*.

## Discussion

This study documents the imaging diagnoses obtained from brain MRI examinations performed at a single academic hospital over 7-years. It provides overview information on lesion prevalence and demonstrates associations between lesion type, patient signalment, and presenting complaint. Such information can assist clinicians with determining which patients to scan and what imaging diagnoses may be expected based on signalment and presenting complaint.

Our results showed that age of dogs significantly differed by imaging diagnosis. The median age of dogs diagnosed with a brain mass was greater than that of dogs with no significant findings and dogs with an imaging diagnosis of AEM were younger than those with a brain mass and NSF. In dogs presenting with seizures, the odds of NSF decreased with each additional year of age, and the odds of a brain mass diagnosis increased with each year of age. Although intracranial neoplasia is typically thought of in older dogs and idiopathic epilepsy (with no significant findings on MRI) and AEM are thought of in younger dogs (10–13), no previous studies to the authors' knowledge have compared the distribution of age among these three groups in a single population. As many of these dogs with different imaging diagnoses had similar presenting complaints (i.e., seizures), our data shows the importance of considering age when interpreting scans and deciding on the patient population to scan. Furthermore, such information may help in owners' decisions to pursue or forego advanced imaging and the substantial associated cost.

The incidence of intracranial neoplasia in all dogs has been reported to be up to 2.6% (14). In our study, 22.36% of all dogs undergoing brain MRI had some form of mass as the most pertinent imaging diagnoses. Exactly 50% of brain masses identified in this study with masses were extra-axial. This is consistent with previous findings that meningiomas, which are extra-axial, are the most common primary canine brain tumor (45%) (14). The frequency of brain mass lesions in dogs was significantly greater in boxers and Boston terriers compared to other breeds. Over 50% of gliomas are reported to occur in brachycephalic breeds including boxers, Boston terriers, English bulldogs, and French bulldogs (14). Although it has been described in the literature that older boxers may be predisposed to intracranial neoplasia (10), this is the first study to the authors' knowledge that analyzed the frequency of canine brain masses by breed and age for all dogs undergoing brain MRI within a single hospital population. In our study, boxer breed and age were found to be significant predictors of brain masses in dogs undergoing brain imaging. Although we do not have histopathologic diagnoses to differentiate neoplastic brain masses from brain masses of other etiologies, we believe that such information still proves valuable. When older boxers are presented with clinical signs such as seizures or mentation changes, an intracranial mass should be considered as a top differential diagnosis. Making the connection between patient presenting complaint and signalment and utilizing this to increase confidence in differential diagnoses has similarly been proven to be important in human medicine. One study showed that prior to MRI being performed, physicians generally already had a high suspicion of disease for patients in whom disease was eventually demonstrated by imaging (15). For example, physicians' median pre-imaging diagnostic confidence was 83% in patients where MRI confirmed an intracranial-space occupying lesion (15).

Dogs with an imaging diagnosis of an intracranial mass commonly presented for seizures. Our analysis showed that >50% of dogs presenting with seizures over the age of 7 years had an imaging diagnosis of a mass (Figure 1). Multiple studies investigating intracranial neoplasia in the dog report the mean age of diagnosis to be 9–10 years (10). For dogs <3 years old presenting with seizures, the predominant imaging diagnosis was NSF. This coincides with the age distribution of canine idiopathic epilepsy described in the veterinary literature, as dogs with idiopathic epilepsy typically present during the ages of 0–3 years (11).

In dogs presenting with seizures, the odds of NSF decreased with increasing age. As previously mentioned, young dogs presenting with seizures frequently had NSF detected on MRI, which could support a diagnosis of idiopathic epilepsy. Taking this into consideration, MRI may have less clinical utility in this population unless other features of signalment or clinical signs increase suspicion of alternate diagnoses. Conversely, given that dogs presenting with seizures have decreased odds of NSF with increasing age, the higher diagnostic yield of MRI in older patients could support the recommendation to this population. By utilizing MRI in this population, more diagnostic information can be provided to help prioritize differential diagnoses and guide subsequent therapeutic treatments. Humans studies have shown the impact MRI findings play in decision making and clinicians will often change their diagnoses and treatments based on MRI findings (15). Given the cost associated with MRI and risks associated with general anesthesia, veterinary clients may want to be better informed on the likelihood of advanced imaging yielding actionable information.

For dogs undergoing brain imaging, there was a significant difference in the frequency of AEM-related lesions between breeds, with the highest incidence in Maltese, Yorkshire terriers, and pugs. Our study also found that increased age reduced the odds of a diagnosis of AEM. These findings are fairly consistent with current literature regarding canine NME (necrotizing meningoencephalitis), GME (granulomatous meningoencephalomyelitis), and MUE (meningoencephalomyelitis of unknown etiology). Canine NME is reported to occur most commonly in young/middle-aged dogs with Pugs, Maltese, and Yorkshire terriers being overrepresented (12). One study looking at MUE found the most common clinical signs encountered were altered mentation, seizures, ataxia, spinal/cervical hyperesthesia, and vestibular signs (16). Our study found that patients with AEM commonly presented for seizures, vestibular signs, and altered mentation.

Two common causes of abnormal mentation/behavior changes in dogs include intracranial neoplasia and canine cognitive dysfunction (CCD), a canine analog of Alzheimer's disease (10, 17–19). Although not looked at in this study, CCD is common in older dogs, particularly those >8 years old (17). In dogs with abnormal mentation in our study, the odds of a brain mass diagnosis increased with each additional year of age. Although numerous studies have discussed behavior change as a presenting complaint associated with an intracranial mass (particularly neoplasia), to the authors' knowledge, no studies have directly correlated these with increasing patient age. While owners of geriatric patients with new onset mentation changes should be aware of CCD, they should also be made aware of the increasing odds of a brain mass with increasing age. Although CCD may be considered a benign disease process with some therapies available to help improve patient quality of life, it is an incurable pathology (17). Therefore, owners may find the results of our study helpful when deciding to pursue the cost and general anesthesia risk associated with MRI in a geriatric patient. Future studies should consider the risk of CCD in relation to increasing age to determine if a similar relationship exists.

There were several study limitations. This was a retrospective, single-institution study that may limit the application of our findings to other institutions. Specifically, having only analyzed data from a single institution, breed bias is inherent in our population. Also, prevalence of lesions will vary for different geographic locations, such as for regions where fungal infections are endemic. The data collection for this project had limitations as imaging reports were written by multiple radiologists and not standardized. Only a single radiologist reviewed these reports retrospectively and decided on the most pertinent imaging diagnosis. It is recognized that such determination is subjective and having multiple radiologists review the reports may have decreased bias. Assessment of each imaging diagnosis was limited because for each diagnosis, multiple histologic diagnoses could account for the finding. General conclusions were drawn from these imaging diagnoses and compared to veterinary literature, but we recognize that “intra-axial masses” for example, cannot be used synonymously with intracranial neoplasia. Furthermore, as histopathologic diagnoses were not available for all cases, only imaging diagnoses and presumed diagnoses were utilized.

## Conclusion

This study documents the imaging diagnoses obtained from brain MRI examinations in canines at a single academic veterinary hospital. These findings provide information on the types and prevalence of diseases observed in the clinical population and reveal correlations between imaging diagnoses, presenting complaints, and features of signalment. This information can help to guide MRI application and justify performing scans in clinical patients.

## Data Availability Statement

The raw data supporting the conclusions of this article will be made available by the authors, without undue reservation.

## Ethics Statement

Ethical review and written consent were not required because this was a retrospective study looking at previously performed MRIs. Therefore, this study was exempt from our institutions requirements for approval by an Ethics Committee. Patient identifying information not included in the study.

## Author Contributions

NW and SS collected the patient data. PC completed the statistical analyses. All authors contributed to the writing, editing of the manuscript, contributed to the article, and approved the submitted version.

## Conflict of Interest

The authors declare that the research was conducted in the absence of any commercial or financial relationships that could be construed as a potential conflict of interest.

## Publisher's Note

All claims expressed in this article are solely those of the authors and do not necessarily represent those of their affiliated organizations, or those of the publisher, the editors and the reviewers. Any product that may be evaluated in this article, or claim that may be made by its manufacturer, is not guaranteed or endorsed by the publisher.
